# The Lowe syndrome protein OCRL1 is involved in primary cilia assembly

**DOI:** 10.1186/2046-2530-1-S1-P87

**Published:** 2012-11-16

**Authors:** V Hernandez, BG Coon, K Madhivanan, D Mukherjee, CB Hanna, I Barinaga-Rementeria Ramirez, M Lowe, PL Beales, RC Aguilar

**Affiliations:** 1 Molecular Medicine Unit, UCL Institute of Child Health, UK; 2 Department of Biological Sciences and Purdue Center for Cancer Research, Purdue University, IN, USA; 3 Faculty of Life Sciences, University of Manchester, UK

## 

Lowe syndrome (LS) is a devastating, X-linked genetic disease characterized by the presence of congenital cataracts, profound learning disabilities and renal dysfunction. Unfortunately, children affected with LS often die early of health complications including renal failure. Although this syndrome was first described in the early 1950s and the affected gene, *OCRL1*, was identified more than 17 years ago, the mechanism by which Ocrl1 defects lead to LS's symptoms remains unknown. Here we show that LS display characteristics of a ciliopathy. Specifically, we found that patients' cells have defects in the assembly of primary cilia and this phenotype was reproduced in cell lines by knock-down of *Ocrl1*. Importantly, this defect could be rescued by re-introduction of WT *Ocrl1* in both patient and *Ocrl1* knock-down cells. In addition, a zebrafish animal model of LS exhibited cilia defects and multiple morphological and anatomical abnormalities typically seen in ciliopathies. Mechanistically, we show that Ocrl1 is involved in protein trafficking to the primary cilia in an Rab8-and IPIP27/Ses-dependent manner. Taking into consideration the relevance of the signaling pathways hosted by the primary cilium, our results suggest hitherto unrecognized mechanisms by which Ocrl1 deficiency may contribute to the phenotypic characteristics of LS. This conceptual change in our understanding of the disease etiology may provide an alternative avenue for the development of therapies.

